# Intrinsic remote conditioning of the myocardium as a comprehensive cardiac response to ischemia and reperfusion

**DOI:** 10.18632/oncotarget.18438

**Published:** 2017-06-12

**Authors:** Noemi Pavo, Dominika Lukovic, Katrin Zlabinger, David Lorant, Georg Goliasch, Johannes Winkler, Dietmar Pils, Katharina Auer, Hendrik Jan Ankersmit, Zoltán Giricz, Márta Sárközy, András Jakab, Rita Garamvölgyi, Maximilian Y. Emmert, Simon P. Hoerstrup, Derek J. Hausenloy, Péter Ferdinandy, Gerald Maurer, Mariann Gyöngyösi

**Affiliations:** ^1^ Department of Cardiology, Medical University of Vienna, Vienna, Austria; ^2^ Department of Anaesthesiology, Medical University of Vienna, Vienna, Austria; ^3^ Center for Medical Statistics, Informatics, and Intelligent Systems (CeMSIIS), Medical University of Vienna, Vienna, Austria; ^4^ Department of Surgery, Medical University of Vienna, Vienna, Austria; ^5^ Molecular Oncology Group, Department of Obstetrics and Gynecology, Medical University of Vienna, Vienna, Austria; ^6^ Department of Pharmacology and Pharmacotherapy, Semmelweis University, Budapest, Hungary; ^7^ Department of Biochemistry, Faculty of Medicine, University of Szeged, Szeged, Hungary; ^8^ Department of Biomedical Imaging and Image-Guided Therapy, Medical University of Vienna, Vienna, Austria; ^9^ Center for MR-Research, University Children’s Hospital Zurich, Zurich, Switzerland; ^10^ Institute of Diagnostic Imaging and Radiation Oncology, University of Kaposvar, Kaposvar, Hungary; ^11^ Swiss Centre for Regenerative Medicine, University of Zurich, Zurich, Switzerland; ^12^ Division of Surgical Research, University Hospital of Zurich, Zurich, Switzerland; ^13^ Clinic for Cardiovascular Surgery, University Hospital of Zurich, Zurich, Switzerland; ^14^ The Hatter Cardiovascular Institute, University College London, London, UK; ^15^ Cardiovascular and Metabolic Disorders Program, Duke-NUS Graduate Medical School, Singapore, Singapore; ^16^ National Heart Research Institute Singapore, National Heart Centre, Singapore, Singapore; ^17^ Yong Loo Lin School of Medicine, National University of Singapore, Singapore, Singapore; ^18^ The National Institute of Health Research, University College London Hospitals Biomedical Research Centre, London, UK; ^19^ Barts Heart Centre, St Bartholomew’s Hospital, London, UK; ^20^ Pharmahungary Group, Szeged, Hungary

**Keywords:** cardioprotection, gene expression, NOGA mapping, ischemia/reperfusion, LV remodelling

## Abstract

We have previously shown that distal anterior wall ischemia/reperfusion induces gene expression changes in the proximal anterior myocardial area, involving genes responsible for cardiac remodeling. Here we investigated the molecular signals of the ischemia non-affected remote lateral and posterior regions and present gene expression profiles of the entire left ventricle by using our novel and straightforward method of 2D and 3D image reconstruction. Five or 24h after repetitive 10min ischemia/reperfusion without subsequent infarction, pig hearts were explanted and myocardial samples from 52 equally distributed locations of the left ventricle were collected. Expressional changes of seven genes of interest (HIF-1α; caspase-3, transcription factor GATA4; myocyte enhancer factor 2C /MEF2c/; hexokinase 2 /HK2/; clusterin /CLU/ and excision repair cross-complementation group 4 /ERCC4/) were measured by qPCR. 2D and 3D gene expression maps were constructed by projecting the fold changes on the NOGA anatomical mapping coordinates. Caspase-3, GATA4, HK2, CLU, and ERCC4 were up-regulated region-specifically in the ischemic zone at 5 h post ischemia/reperfusion injury. Overexpression of GATA4, clusterin and ERCC4 persisted after 24 h. HK2 showed strong up-regulation in the ischemic zone and down-regulation in remote areas at 5 h, and was severely reduced in all heart regions at 24 h. These results indicate a quick onset of regulation of apoptosis-related genes, which is partially reversed in the late phase of ischemia/reperfusion cardioprotection, and highlight variations between ischemic and unaffected myocardium over time. The NOGA 2D and 3D construction system is an attractive method to visualize expressional variations in the myocardium.

## INTRODUCTION

Metabolism and gene expression patterns are changing intensively in the ischemic regions of an infarcted heart, but also remote heart regions respond to the injury quickly. We have recently shown that both acute and chronic ischemia alters the molecular signals of the ischemia non-affected, but adjacent regions, termed as intrinsic remote conditioning against adverse left ventricular (LV) remodeling [1, 2].

Conditioning of the heart against ischemic injury is one of the most potent mechanisms to prevent the heart from ischemic damage [3–7]. Single or repetitive brief intervals of ischemia and reperfusion (r-I/R) induce cardioprotective effects against a subsequent ischemic insult. The protective effect is characterized by two time windows: Early effects last a few hours, and are conferred by the rapid release of transmitter molecules such as bradykinin and prostaglandins. The second window of protection (SWOP) is mainly relying on transcriptional regulation, mediated by activation of kinases and transcription factors, and subsequent effects of proteins generated *de novo*. The transcriptional changes are initiated quickly after r-I/R, and are fully effective about one to three days later. Although clinical translation of ischemic preconditioning is difficult to achieve for practical reasons, elucidation of the underlying mechanisms might lead to identification of potential modulating agents and molecular targets for the development of novel therapeutic strategies [7].

For the investigation of molecular changes in the myocardium in heart diseases, precise sampling of tissue sections, including documentation, is necessary for facilitating understanding of regional variations between directly affected and remote tissue [8, 9]. In many rodent models and experiments, the entire heart is often analyzed as a whole due to its small size. In large animals, the affected heart tissue, and usually a randomly selected sample of remote heart tissue (often used for “normal” control sample) is used for analyses by histology, or for gene expression or protein abundance. Exact locations of sampling are usually not reported, and the potential impact of the sampling locations on the analysis outcome is often neglected.

The three-dimensional NOGA^®^ (Biologics Delivery Systems, a Johnson & Johnson company, Irwindale, CA, USA) mapping system is equipped for simultaneous measurement of electrical and mechanical activities of the myocardium and distinguishes between viable and non-viable myocardium [10]. The NOGA and the CARTO systems have the same principles to construct 2D and 3D real-time display of the myocardial viability, wall motion and electrical activity, and are currently the only real time 3D imaging technologies available for the clinics. In addition the NOGA system can be used for guided intramyocardial injections of biologicals.

Here, we show the use of the NOGA system, employed as a transcardial mapping *in vitro*, for precise documentation of locations of tissue samples, and for creating a map of gene expression levels. By replacing the voltage values of the NOGA map by the respective fold changes of qPCR quantification, it is possible to build color-coded two- or three-dimensional images, which aptly visualize spatial gene expression. In order to demonstrate the utility of this approach, we determined and compared gene expression values in pig hearts with repetitive ischemia and reperfusion in a time-window of 5h and 24h after I/R burden, compared to controls.

## RESULTS AND DISCUSSION

**Figure 1:**
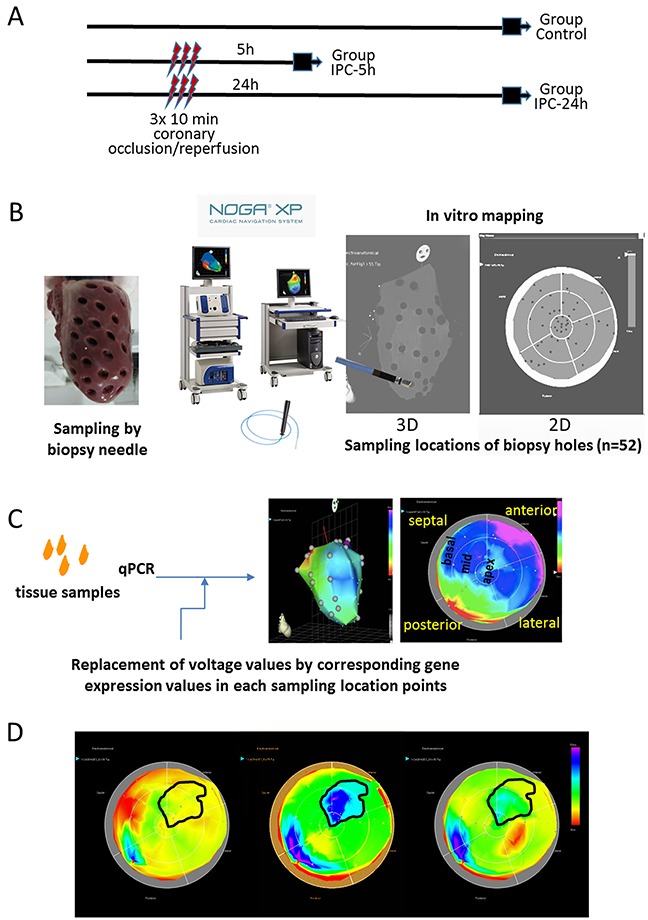
Study design and principle of NOGA-guided image-omics **(A)** Timeline of the protocol and the three groups. Gene expression profiles of the whole LV was determined either without intervention (Group Control), or 5h (Group I/R-5h) or 24h (Group I/R-24h) after 3×10 min I/R by repetitive inflation/deflation of an intracoronary balloon placed in the mid part of the porcine left anterior descending coronary artery. **(B)** Method of the myocardial sampling: the explanted heart (without the right ventricular part) was placed on a 50 mL Falcon tube, and 52 myocardial biopsies were collected with a skin biopsy needle with an equal distribution of the sampling locations throughout the entire left ventricle. The biopsy samples were labelled in accordance to the biopsy location, and transferred immediately into RNALater. **(C)** Schematic illustration of the image-omics of the gene expression maps. Using mapping principles of the myocardial viability map, sampling locations were detected in *in vitro* epicardial (surface) NOGA-mapping, using the location point record principles. The voltage values recorded by NOGA in the distinct locations were replaced by the respective values of fold changes in gene expressions gathered from the excised tissue samples. **(D)** Delineation of the ischemic and remote areas on the 5h polar map images with transposition of the same area onto the control and 24h polar maps. Gene expression values of the corresponding ischemic and remote areas of the animals in control, 5h and 24h groups were collectively compared for statistical evaluations.

Five and 24h after induction of r-I/R by three cycles of 10 min percutaneous balloon occlusion/deflation of the mid left anterior descending coronary artery, the hearts were explanted. For generation of gene expression images, 52 tissue samples were collected from the explanted pig hearts and utilized the NOGA system for defining the precise location of each sample (Figure 1). Gene expression for each tissue sample was gathered by qPCR. Then, the relative expression values were entered in place of the voltage or local activation data in the NOGA system. This workflow demonstrates that data visualization using the NOGA system is straightforward. The intrinsic color coding and 2D and 3D capabilities allow for simple and attractive data visualization and thus identification of regions with high or low expression levels. In particular, the sampling location of the remote myocardium is important for the gathered molecular data, as for some of the examined genes their expression differs significantly within unaffected myocardial tissue sections. However, it must be noted that expression in basal tissue segments (outer regions in the bulls-eye view) needs to be interpreted with some caution, since the tissue composition differs (mixture of fibrotic and muscular tissue).

We collected a total of 52 tissue samples for each pig heart and compared the individual expression levels of seven genes of interest – HIF-1α, caspase-3, transcription factor GATA4, myocyte enhancer factor 2C (MEF2c), hexokinase 2 (HK2), clusterin (CLU) and excision repair cross-complementation group 4 (ERCC4) between control animals and pigs that underwent r-I/R (Figure 1). The gene selection was based on earlier NGS analyses of ischemic and remote tissue areas, and we primarily selected genes with currently incompletely elucidated functions in ischemic injury (except for HIF-1α) [2]. Two distinct time points were investigated; five hours after r-I/R, to examine quick transcriptional regulation, and 24 h after r-I/R, to gain information on transcriptional changes relevant for later, sustained cardioprotection, (SWOP). After filling the values obtained by qPCR to the NOGA software, two- and three-dimensional visualizations were readily obtained (Figure 1).

**Figure 2:**
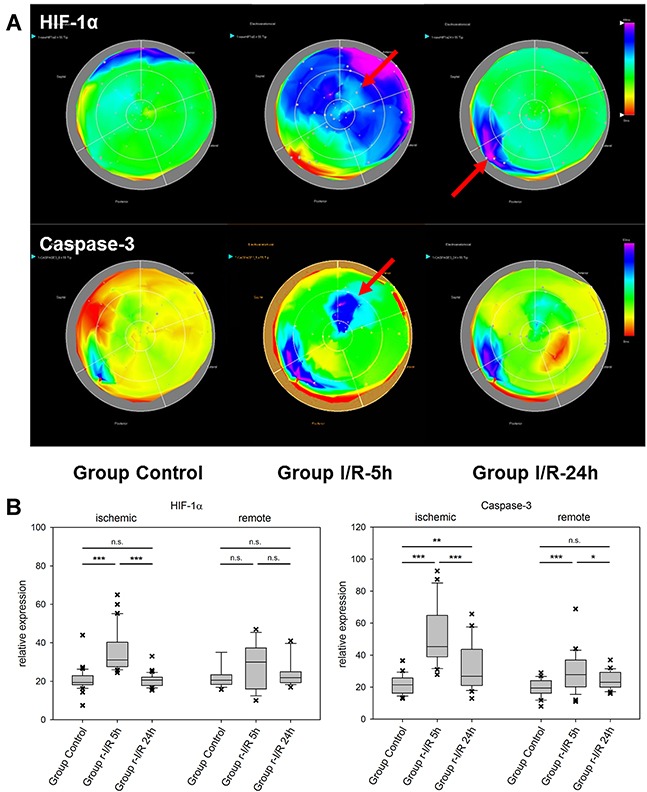
Spatiotemporal 2D bulls-eye display of HIF-1α, and caspase-3 gene expression of the entire left ventricle after repetitive ischemia/reperfusion (r-I/R) **(A)** Time-dependent presentation of the different gene expression patterns of HIF-1α, and caspase-3, of the LV of animals in groups control, I/R-5h and I/R-24h after repetitive (3 times) 10 min I/R without consecutive myocardial infarction. Temporary overexpression of HIF-1α and caspase-3 at 5h (red arrow) was detected. Expression levels of HIF-1α and caspase-3 were reduced to baseline levels after 24h, except for part of the remote area with HIF-1α upregulation. Pink and blue colors represent up-regulation of genes; green represents baseline values, while yellow and red areas show down-regulation of the respective genes. **(B)** Boxplots of values of the ischemic and remote zone. *: p<0.05, **: p>0.01, ***: p<0.001. n.s.: not significant.

In ischemic preconditioning, HIF-1α is essential for protection from consequent ischemia in both the acute and delayed (SWOP) phases of protection [11, 12]. HIF-1α is a powerful transcriptional regulator and among its target genes are VEGF, EPO, inducible nitric oxide synthase (iNOS), and angiopoetin 1 and 2. The regulated genes and the functional consequences are to a certain degree cell-type dependent [13] and include increase of angiogenesis, vascular remodeling, and glucose metabolism [12]. In pig hearts, HIF-1α was upregulated in ischemic and parts of the remote myocardium after 5h (Figure 2). At 24h, we observed unchanged HIF-1α expression compared to controls in those heart regions, but upregulation in the remote zone that had shown unchanged expression levels at the earlier time point. This expressional pattern indicates an initially strong activation of HIF-1α after r-I/R primarily in the directly affected tissue, which recedes after 24h. Interestingly, transcriptional upregulation of HIF-1α seems to be restricted to the early phase, but it is documented that HIF-1α protein is additionally stabilized after preconditioning [14]. Thus the downstream targets of HIF-1α are active also in the late phase of cardioprotection.

**Figure 3:**
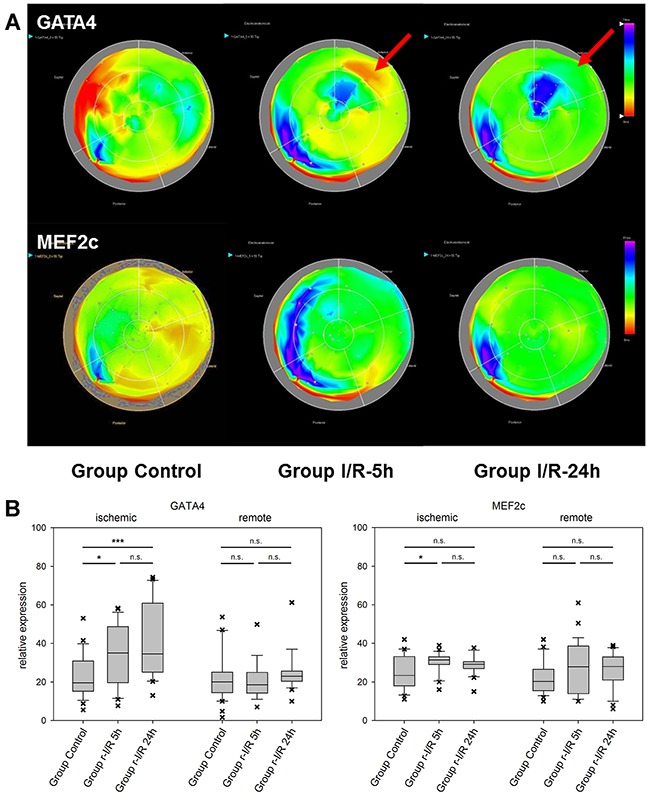
Spatiotemporal 2D bulls-eye display of GATA4 and myocyte enhancer factor 2C (MEF2c) gene expression of the entire left ventricle after repetitive ischemia/reperfusion (r-I/R) **(A)** Time-dependent presentation of the different gene expression patterns of GATA4 and MEF2c of the LV of animals in groups control, I/R-5h and I/R-24h after repetitive (3 times) 10 min I/R without consecutive myocardial infarction. Mildly increasing upregulation of GATA-4 in the ischemic area at 5 and 24h (red arrows) was detected. Only mild upregulation of MEFC2 in the ischemic area was encountered. Pink and blue colors represent up-regulation of genes; green represents baseline values, while yellow and red areas show down-regulation of the respective genes. **(B)** Boxplots of values of the ischemic and remote zone. *: p<0.05, **: p>0.01, ***: p<0.001. n.s.: not significant.

The effector caspase-3 is activated in both the intrinsic and extrinsic apoptosis pathways. It is an important apoptosis mediator in myocardial infarction., Caspase-3 is detected in human serum after an infarct, due to escaping into the bloodstream following myocardial cell death [15]. Its mRNA levels were substantially elevated in ischemia-affected myocardium (Figure 2) 5h after r-I/R, but were reduced nearly back to baseline levels at the later time point. In remote areas, a lower extent of caspase-3 upregulation resulted at 5 h and transcription receded back to control levels 24 h after ischemia. These data indicate that a certain degree of apoptosis occurs shortly and temporarily after ischemia only in directly affected tissue. We also detected a region with increased expression of caspase-3 in the basal inferoseptal area. Since similar upregulation was found for other analyzed genes (Mef2c, GATA4, and HIF-1α), this may indicate a local inflammatory process. However, expressional data in the basal areas needs to be interpreted with some caution. The stress-associated transcription factor GATA4 is essential for cardiac gene expression in general and modulates adaptive responses after injury [16]. It confers regenerative effects and is critical for the regenerative capability of neonatal mice hearts [17]. Together with transcription factors Mef2c and Tbx5 (the combination of the three factors is termed GMT), it was reported to be an essential factor in cardiac reprogramming [18, 19]. In injured heart tissue, GATA4 was increasingly upregulated over 24h after r-I/R. Similarly to Caspase-3, its upregulation was limited to the infarcted area (Figure 3). The time-sequence of up regulation suggests that expression of pro-survival genes such as GATA4 follow an initial activation of pro-apoptotic genes such as caspase-3. In contrast, the expression level of the cardiac transcription factor MEF2c was found to be largely unchanged (Figure 3). This indicates that transcriptional activation of MEF2c is not playing a major role in SWOP induced by r-I/R.

**Figure 4:**
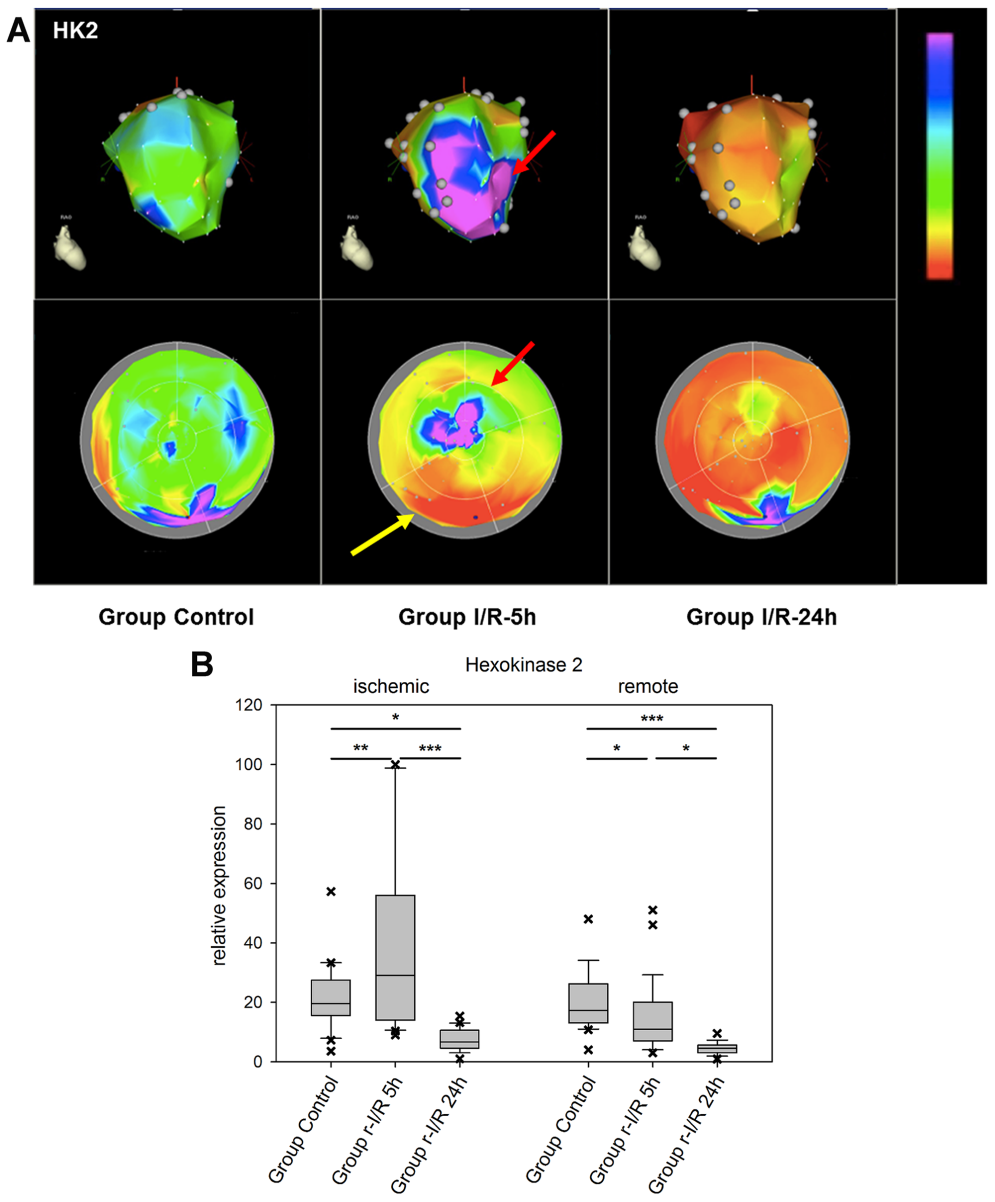
Image-omics (2D and 3D modeling) of the repetitive ischemia/reperfusion (I/R) induced gene expression pattern of hexokinase 2 (HK2) **(A)** Representative 3D (top row) and 2D bulls-eye maps (bottom row) of the left ventricle (LV) showing the expression patterns of HK-2. Marked upregulation of HK2 was found in the ischemia-affected apical myocardial region and the border zone with concomitant downregulation in the remote myocardial area (yellow arrow) at 5h. At 24h, HK2 expression was severely downregulated in all myocardial regions. **(B)** Boxplots of values of the ischemic and remote zone. *: p<0.05, **: p>0.01, ***: p<0.001.

Hexokinase-2 is an important enzyme in the glucose metabolism, namely the phosphorylation of glucose as one of the initiating steps of glycolysis. In addition, HK-2 has been shown to be protective against oxidative stress and to attenuate the production of ROS [20]. Mitochondrial binding of HK-2 promotes cell survival. Decreased levels of HK-2 after r-I/R resulted in altered remodeling with higher rates of cell death and fibrosis and lower angiogenesis [21]. In mice, HK-2 knockdown exaggerated cardiac hypertrophy after induction of pressure overload [22]. In r-I/R pig hearts, spatial analyses of expression showed short-term up-regulation of HK-2 in the ischemic area only, with a pronounced down regulation in some remote areas 5h after r-I/R and in all heart areas 24h after r-I/R (Figure 4 and Supplementary Videos 1–3). The quick up regulation after ischemic injury may be indicative of an activation of pro-survival signaling, and the reduction in all heart areas after 24 h is likely to have an important impact of cell energy metabolism, but also ROS production, and cardiomyocyte survival, and indicates an onset of the molecular processes leading to cardiac remodeling.

**Figure 5:**
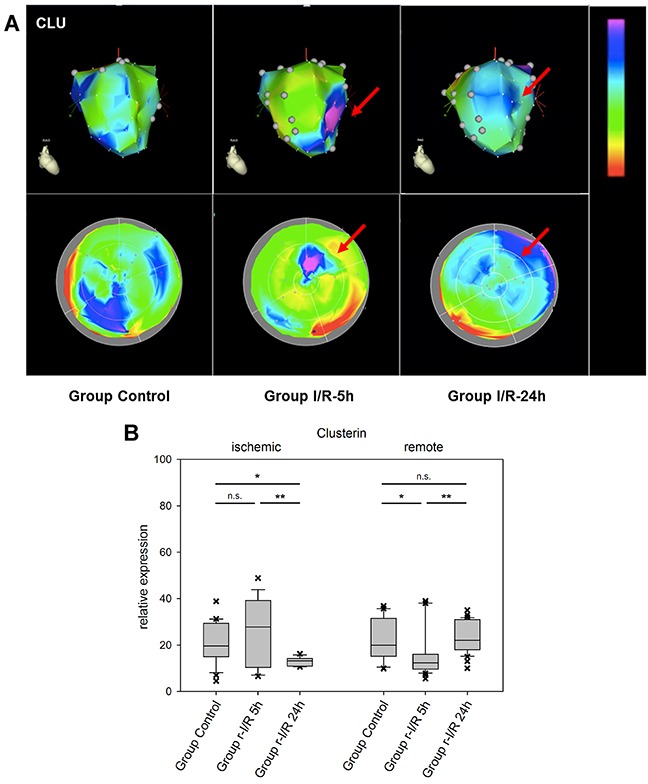
Image-omics (2D and 3D modeling) of the repetitive ischemia/reperfusion (I/R) induced gene expression pattern of clusterin (CLU) **(A)** Representative 3D (top row) and 2D bulls-eye maps (bottom row) of the left ventricle (LV) showing the expression pattern of CLU. Moderate up-regulation in part of the ischemic zone was detected at 5h (red arrow), with a high degree of variation within the remote myocardium. **(B)** Boxplots of values of the ischemic and remote zone. *: p<0.05, **: p>0.01, ***: p<0.001. n.s.: not significant.

The cytoprotective chaperone clusterin/apolipoprotein J (CLU) is produced and secreted in response to stress signals. Its plasma levels are increased in several disorders, including neurodegenerative diseases and neoplasms, but also atherosclerosis and myocardial infarction [23]. Interestingly, CLU was recently reported to be associated with survival in patients with heart failure [24]. In the myocardium, CLU protects against apoptosis, modulates matrix metalloproteinase expression and stimulates angiogenesis. In a complex with the proteohormon leptin, it binds to the leptin receptor, which results in transcriptional activation of intracellular pathways including the JAK/STAT pathway [25]. We encountered an increase in CLU expression already 5h after ischemic injury in the apex part of the affected tissue. In remote areas, considerable variation was found, with downregulation at the earlier time point in one zone and a considerable elevation of expression in another part of the remote zone 24h after r-I/R (Figure 5). These data are an indication that CLU plays a role in restoring cell function, cardioprotection and might be an important mediator of intrinsic remote conditioning.

**Figure 6:**
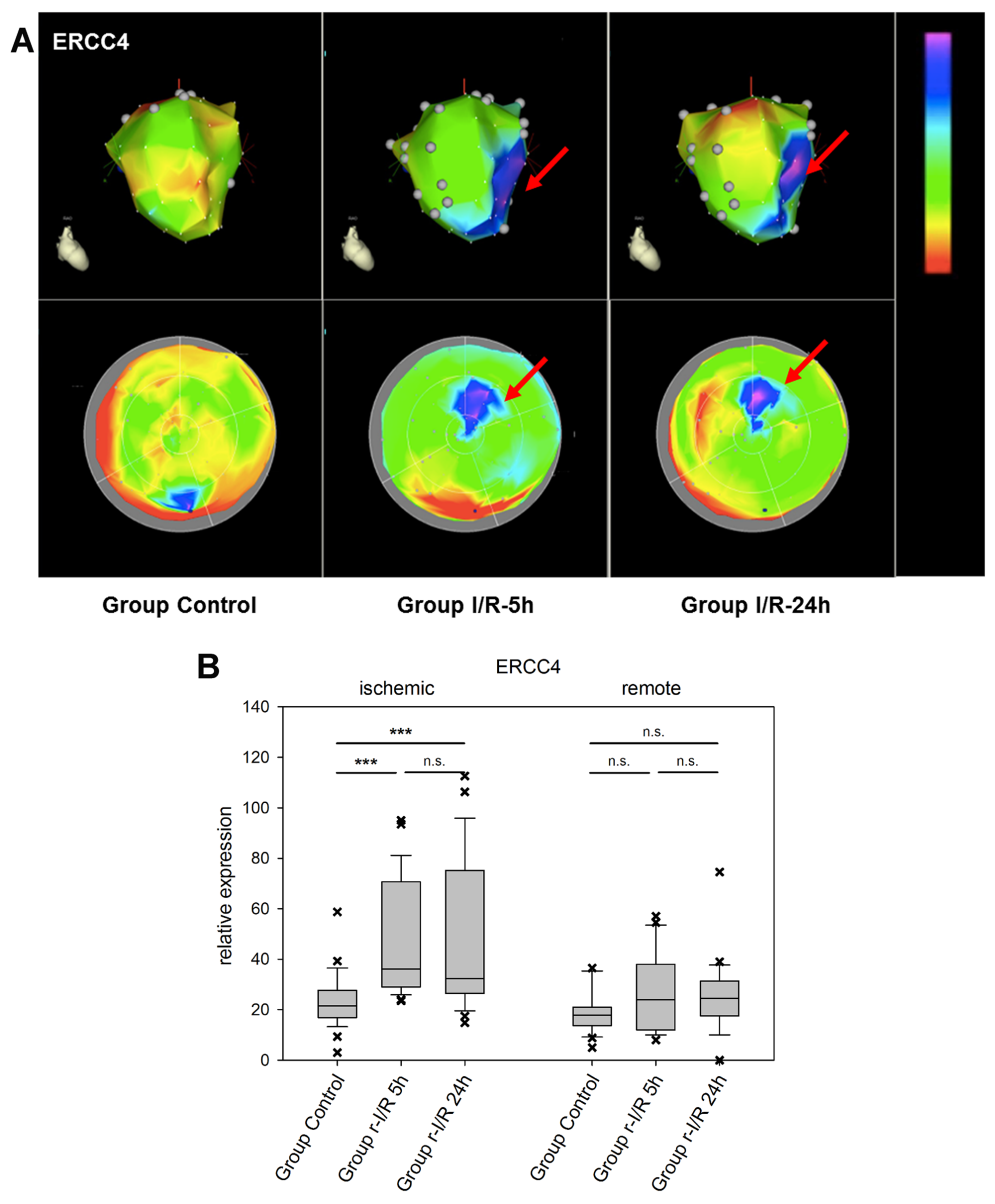
Image-omics (2D and 3D modeling) of the repetitive ischemia/reperfusion (I/R) induced gene expression pattern of excision repair cross-complementation group 4 (ERCC4) **(A)** Representative 3D (top row) and 2D bulls-eye maps (bottom row) of the left ventricle (LV) showing the expression pattern of ERCC4. Moderate up-regulation is shown in the ischemic zone at both 5h and 24h (red arrows), with little changes between the two time points. **(B)** Boxplots of values of the ischemic and remote zone. *: p<0.05, **: p>0.01, ***: p<0.001. n.s.: not significant.

The excision repair cross-complementation group 4 (ERCC4/XPF) gene is a subunit of the ERCC1/XPF endonuclease which has a function in repair of DNA damage and stress response [26]. It cleaves nucleic acids specifically at junctions between double- and single stranded DNA and is a component of the machinery for nucleotide excision repair, and others. We identified ERCC4 to be upregulated after r-I/R in an NGS dataset and our spatiotemporal analysis shows that the up regulation is focused on the tissue directly affected by ischemia (Figure 6). While a deficiency of ERCC1/ERCC4 has been linked to carcinogenesis and cancer progression, its role in the myocardium is currently unknown. The upregulation of ERCC4 might be a consequence of cell and nucleic acid damage.

Exploiting the 3D reconstruction technology of the NOGA endocardial (here epicardial) electroanatomical mapping system, we demonstrate a new and straightforward methodology with which to display gene expression patterns in 2D and 3D, without the need for an extensive bioinformatics background or training. The image-omics that we present of the ischemic preconditioned heart integrates genomic data with biomedical imaging. This facilitates exploration and visualization of relevant gene expression patterns that underlie the SWOP. These data provide biological insight into cardioprotective mechanisms that are essential for better understanding of the complexity of I/R injury.

As reported earlier, the NGS-based analysis of global gene expression patterns in ischemic, border, and remote zones [2] showed distinct changes of several pathways and a number of genes that were previously not linked to ischemic preconditioning. The gene expression patterns show a quick onset of regulatory response after r-I/R in the directly affected tissue with up-regulation of genes involved with stress response and apoptosis. The more detailed spatial expression patterns reported here corroborate the NGS results and also highlight the role of intrinsic remote conditioning: that remote heart areas (unaffected by ischemia) can have varying expression levels of genes playing important roles in cardioprotection and prevention of adverse left ventricular remodeling. For investigating mechanisms such as cardiac remodeling on the molecular level, it may thus be advisable to harvest tissue sections from a few spots of the remote zone. More importantly, we show that the NOGA system is particularly useful, yet very facile to use for constructing 2D and 3D representations of expression patterns. Since externally gathered values of biopsies that were taken with the system can be simply entered into the software, this approach can likewise be employed for any other readout, for example from histological analysis or from proteomics data.

### Limitation

We have not compared the transcriptomic expressions levels of each sampling location between control and 5h or 24h samples, because one sample of a certain location does not represent the global myocardial response to ischemic burden. It is possible to quantify segmental gene expression levels by using the NOGA 9-segment model, however, this method is not standardized. Additionally, the ischemic or borderline ischemic myocardial areas cannot be delineated into pre-defined 9 segments due to overlap of ischemic and non-ischemic areas.

We used also the 17-segment model for segmental gene expression quantification, according to the recommendation of the AHA for segmentation of the LV by using echocardiography and cardiac MRI. However, this method has some limitations. Firstly, the principles of the 3D and polar map reconstructions of the MRI and NOGA images as well as the point sampling method differ [10]. Secondly, analyzing an average of three samples in each of the 17 segments precludes a robust statistical evaluation. Extended sampling of at least 90 biopsy samples, corresponding to 5 or more samples in each segment would be required.

## CONCLUSION

Using 2D and 3D visualization (2D and 3D image-omics) of temporal and spatial gene expression maps of the heart, we demonstrate that r-I/R stimuli provoke distinct alterations in gene expression profiles in different regions of the myocardium. We employed a clinically relevant closed-chest pig model to highlight transcriptional regulations induced by r-I/R. The analysis of multiple tissue samples at several time points with the novel methodology described herein increases our understanding of r-I/R mechanisms. The reported data indicate a short term stress response, which is followed by prolonged expressional alterations, including transcriptional regulators and survival signals, which are essential for the second window of cardioprotection after ischemic preconditioning in the ischemia affected but also in the non-affected myocardial areas.

## MATERIALS AND METHODS

### Ethical statement

Animal investigations were carried out in accordance with the “Position of the American Heart Association on Research Animal Use,” as adopted by the AHA on November 11, 1984. The study was approved by the Ethics Committee on Animal Experimentation at the University of Kaposvar, Hungary. The study design is displayed in Figure 1A. The study corresponds to the ARRIVE guidelines [27].

### Porcine model of repetitive ischemia/reperfusion

Domestic pigs (male, 15 kg, n=20, randomized into r-I/R[5h], n=6, r-I/R[24h], n=6, and sham operated controls, n=8) underwent cardiac catheterization under general anaesthesia. The r-I/R protocol consisted of three repetitive cycles of 10 min I/R via percutaneous balloon occlusion and deflation in the mid left anterior descending coronary artery (LAD) as described previously [2].

Briefly, the pigs received an intramuscular injection of 12 mg/kg ketamine hydrochloride, 1 mg/kg xylazine and 0.04 mg/kg atropine, with inhalation anesthesia with isoflurane and O_2_. After reaching deep anesthesia, pigs were intubated and the anaesthesia was continued with an anesthetic gas mixture of 1.5-2.5 vol% isoflurane, 1.6-1.8 vol% O_2_ and 0.5 vol% N_2_O. A 6F introduction sheath (Medtronic Inc, Minneapolis, MN) was placed into the right femoral artery followed by intra-arterial administration of unfractionated heparin (200 IU/kg). A 6F coronary catheter (Medtronic Inc, Minneapolis, MN) was placed into the abdominal aorta and selective angiography of the left coronary arteries was performed. A guidewire (Medtronic Inc, Minneapolis, MN) and then a coronary balloon dilation catheter (2.75 mm diameter, 12 mm length; Medtronic Inc, Minneapolis, MN), were placed into the left anterior descending coronary artery below the origin of the second diagonal branch. In the r-I/R groups (n=12), coronary occlusion was performed with 6 atm inflation pressure. Coronary angiography was done by injecting non-ionic contrast media (Takeda, Zürich, Switzerland) to monitor occlusion and reperfusion of the coronary artery.

### Myocardial sampling method

At either 5 h or 24 h after the procedure (Figure 1), the animals were sacrificed, and hearts were explanted and the right heart part was removed. The entire LV was fixed on a 50 mL Falcon tube, and 52 myocardial biopsy samples were excised using a skin biopsy needle (Acu-Punch, Acuderm, Fort Lauderdale, FL). Care was taken for equal distribution of the samples throughout the entire LV according to anatomical landmarks. The samples were uniquely labelled according to a predefined scheme. The collected samples were immediately stored in RNALater.

### *In Vitro* NOGA-mapping for image-omics

To enable 2D and 3D displays of the changes in gene expression in the LV, the sampling locations were determined by *in vitro* surface NOGA mapping directly after sample collection from the explanted heart. The NOGA mapping principles and technique have been described previously [10]. Briefly, using an ultralow magnetic field and a NogaStar® catheter with a magnetic tip (Johnson & Johnson, Diamond Bar, California), the NOGA system displays the heart showing the location of the catheter tip in 3D, and measures the actual electrical signals. In contrast with the real-time use of this system to illustrate location of the catheter tip in relation with viability of that area, for *in vitro* mapping we used only the sampling locations (Figure 1B).

Briefly, the heart (mounted on Falcon tube) with the biopsy holes were then put on the angiographic table simulating the heart *in vivo* position, and the heart was subjected for *in vitro* NOGA anatomical surface mapping. The NOGA mapping catheter was placed exactly on the epicardial surface of each biopsy hole, recording the sampling location coordinates in the NOGA system, assigning each sample allocation to x, y and z Cartesian, and rho and theta polar map coordinates. In this way, the heart with sampling holes (NOGA location points) has been visualized in the 2D and 3D NOGA imaging map (Figure 1C).

### qPCR

Tissue sections in RNAlater were stored at −80°C until performing RNA isolation. RNA was isolated from the samples using column based extraction (RNeasy, Qiagen, Germany). RNA concentrations were determined using a Nanodrop spectrophotometer (Thermo Fisher) and 500 ng of each sample were reverse transcribed with random hexamer primers to cDNA according to the manufacturer’s protocol (Qiagen). Gene expressions were quantified on an Applied Biosystems 7500 Real-Time PCR System (Life Technologies, USA) using Sybr Green Master Mix (Qiagen) with primers listed in Table 1. Relative expression values were calculated using the ΔΔCt method with normalization to the geometric mean of the three reference genes GAPDH, HPRT1, and PPIA. For each qPCR run, a calibration curve derived from five dilutions (1:8 each) of the same sample was generated for normalization across the entire analysis.

**Table 1:** Primer sequences and amplicon lengths for qPCR

Gene	Forward primer	Reverse primer	Ampliconlength (bp)
Caspase-3	GGGATTGAGACGGACAGTGG	TGAACCAGGATCCGTCCTTTG	136
Clusterin	CATGAAGTTCTACGCGCGTG	AGTAGAAGGGGGAGCTCTGG	92
ERCC4	ATGGGAAGCACTGACCGAAG	GAACACGTCCTGTCGTCACT	114
GATA4	AGAAAACGGAAGCCCAAGAAC	CCACACTGCTGGAGTTGCTG	109
HK-2	CAGCAGAACAGCCTGGATGA	GGATGGCTTCCTTCAGCAGT	106
MEF2c	TAACATGCCGCCATCCGCCC	ATCCTCTCGGTCGCTGCCGT	151
GAPDH	TCCACCCACGGCAAGTTCCAC	ATGTTGGCGGGATCTCGCTCCT	104
HPRT1	CCCAGCGTCGTGATTAGTGA	ATCTCGAGCAAGCCGTTCAG	131
PPIA	GTCTTCTTCGACATCGCCGT	TCCTTTCTCCCCAGTGCTCA	120

### Image-omics of the gene expression in 2D and 3D

NOGA-derived quantitative viability values of the recorded locations were manually replaced with values of fold changes in gene expressions. Since the NOGA voltage map cannot display negative values, the minimal (maximal down regulation) and maximal (maximal up-regulation) values of expression of each gene were assigned to a scale of 0-100. The basal posterior region value was chosen as basic reference point (value of around 20 for almost all genes). All gene expression intensities derived from the qPCR were converted to this 0-100 scale. The color maps thus show downregulation (min value of 0) in red, yellow for the reference value of the control animals and blue and pink for upregulated genes (max count on 100 scale).

### Statistics

Myocardial ischemic areas at the 5h polar map images separate of each gene maps were delineated, and transposed to the polar maps of the corresponding control and 24h polar map images. Mean values of the gene expression values within and outside this area were assigned to ischemic or remote areas, respectively. The gene expression of the control, 5h and 24h ischemic and remote areas were compared by using ANOVA with Holm-Sidac post-hoc tests.

Additionally, the polar maps containing the 52 location points with the measured qPCR values were divided into 17 segments, similar to the echocardiography and cardiac MRI polar map segmental display, according to the recommendation of the American Heart Association Writing Group on Myocardial Segmentation and Registration for Cardiac Imaging [28]. The mean values of the expression of genes of interest in each 17 segments were calculated and the values were compared to the baseline qPCR values of the same segment. Since each segment contained 3 measured values in average, a direct statistical comparison was not justified. However, where the difference between the control value and 5h or 24h value of the comparative segments was larger than the 2xSD of the mean values of both segments, this difference was considered biologically relevant and displayed in red color in the 17-segment polar map display (Supplementary Figures 1-7).

## SUPPLEMENTARY MATERIALS FIGURES AND VIDEOS









## Abbreviations

AMI: acute myocardial infarction
CLU: clusterin
DPP4: dipeptidyl peptidase 4
EPO: erythropoetin
ERCC4: excision repair cross-complementation group 4
GATA4: transcription factor binding to nucleotide sequence GATA
GMT: GATA4, MEF2c and Tbx5
HIF-1α: hypoxia-inducible factor 1α
HK-2: hexokinase-2
JAK: Janus kinase
MEF2c: myocyte-specific enhancer factor 2C
r-I/R: repeated ischemia and reperfusion
RISC: reperfusion injury salvage kinase
ROS: reactive oxygen species
SAFE: survivor activating factor enhancement
STAT: signal transducer of activation and activator of transcription
VEGF: vascular endothelial growth factor
