# Supplementary material for: Intrinsic remote conditioning of the myocardium as a comprehensive cardiac response to ischemia and reperfusion
Source: Oncotarget. 2017 Jun 12;8(40):67227–40. doi: 10.18632/oncotarget.18438 (PMC5620169; doi:10.18632/oncotarget.18438)
Supplement: Supplementary file 1 [file oncotarget-08-67227-s001.pdf]

Intrinsic remote conditioning of the myocardium as a comprehensive cardiac response to ischemia and reperfusion

SUPPLEMENTARY MATERIALS

Segmental displays of gene expressions according to cardiac MRI polar map views

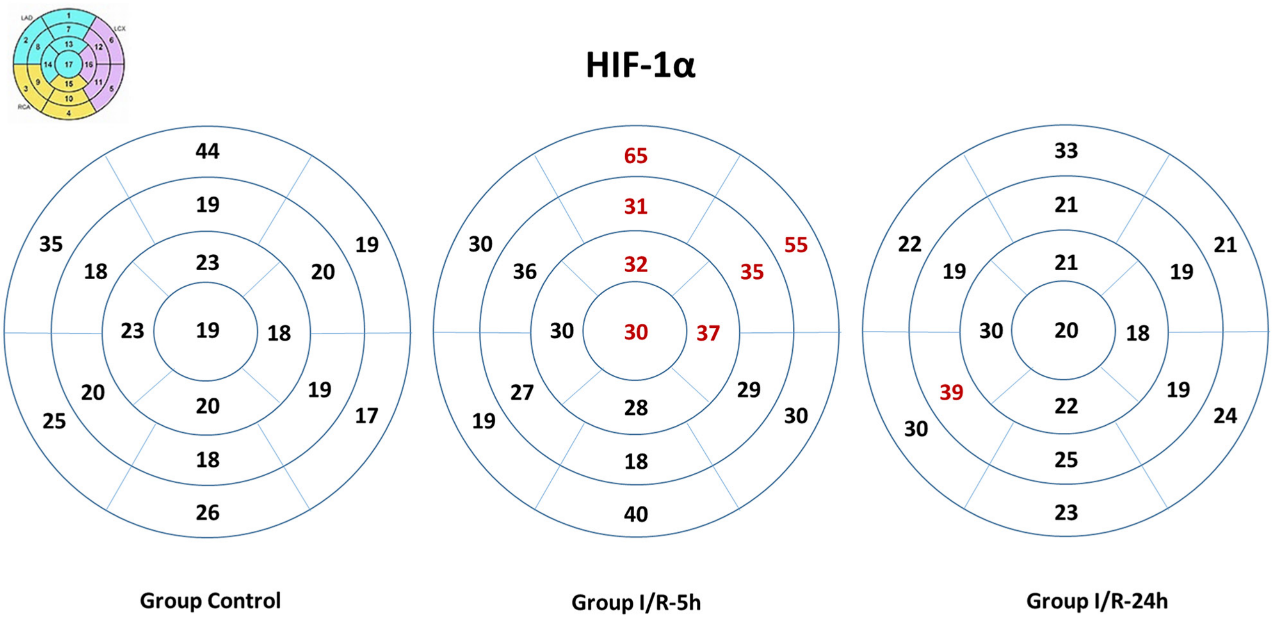

Supplementary Figure 1: Polar map view of relative gene expression values of HIF-1α. Values with higher expression (differences > 2x s.d.) than the control group in the corresponding segment are red.

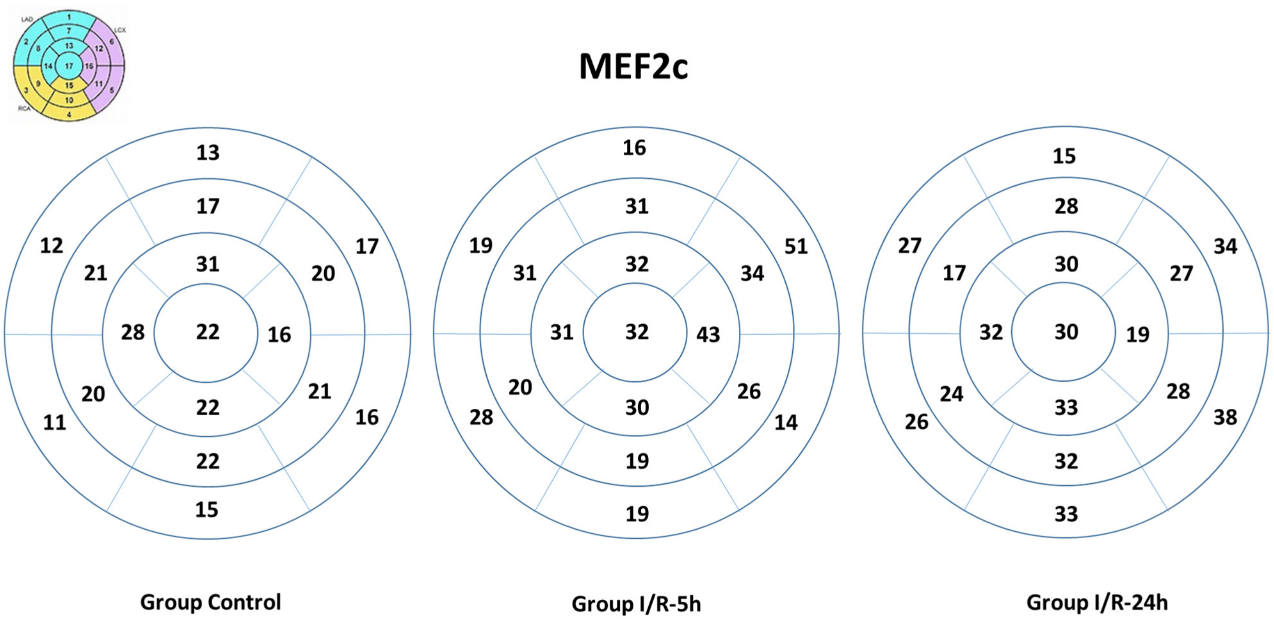

Supplementary Figure 2: Polar map view of relative gene expression values of MEF2c. No significant differences to the controls were found at either time point.

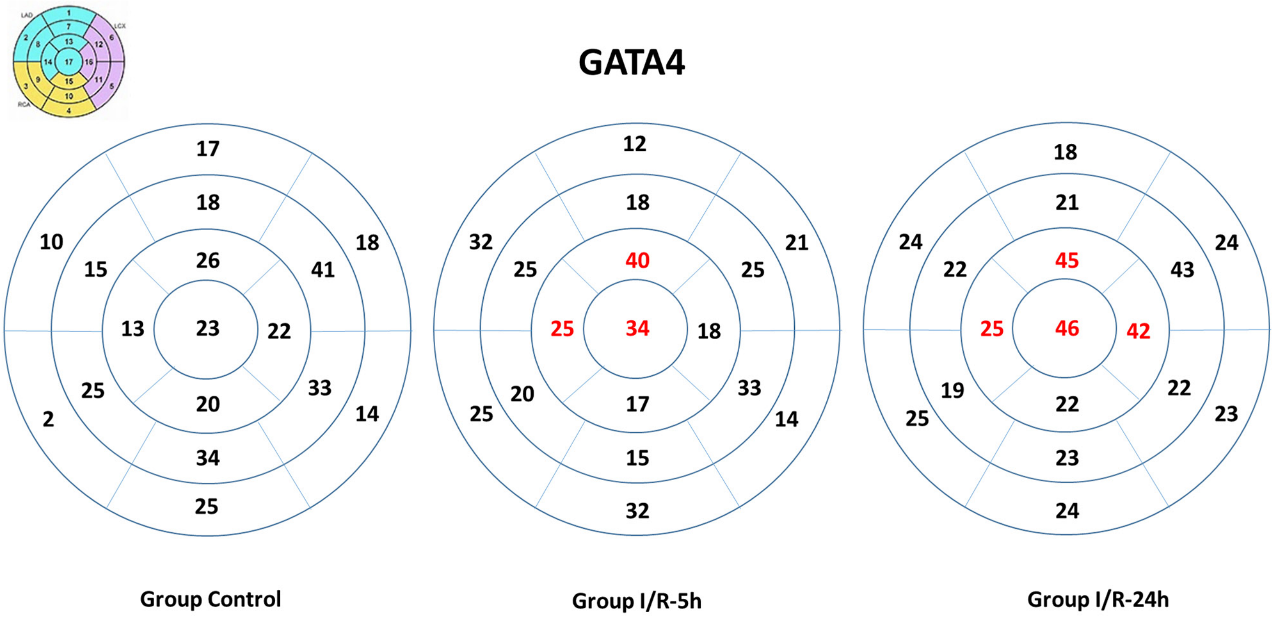

Supplementary Figure 3: Polar map view of relative gene expression values of GATA4. Values with higher expression (differences > 2x s.d.) than the control group in the corresponding segment are red.

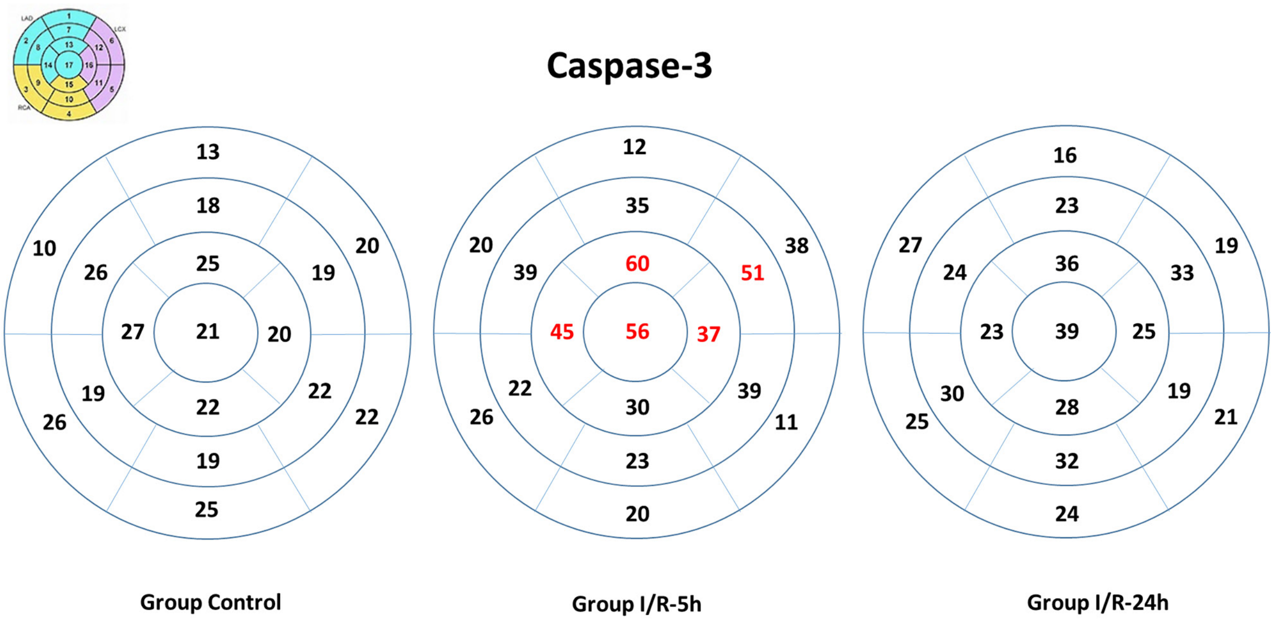

Supplementary Figure 4: Polar map view of relative gene expression values of caspase-3. Values with higher expression (differences > 2x s.d.) than the control group in the corresponding segment are red.

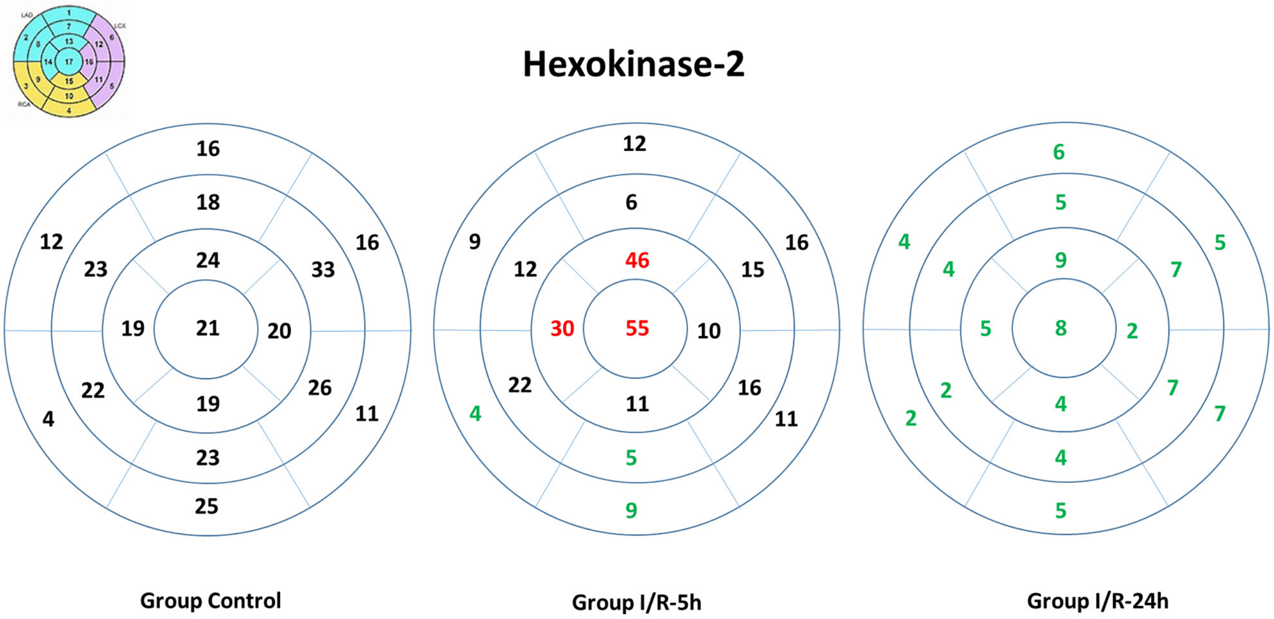

Supplementary Figure 5: Polar map view of relative gene expression values of hexokinase-2. Values with higher expression (differences > 2x s.d.) than the control group in the corresponding segment are red, those with lower expression are green.

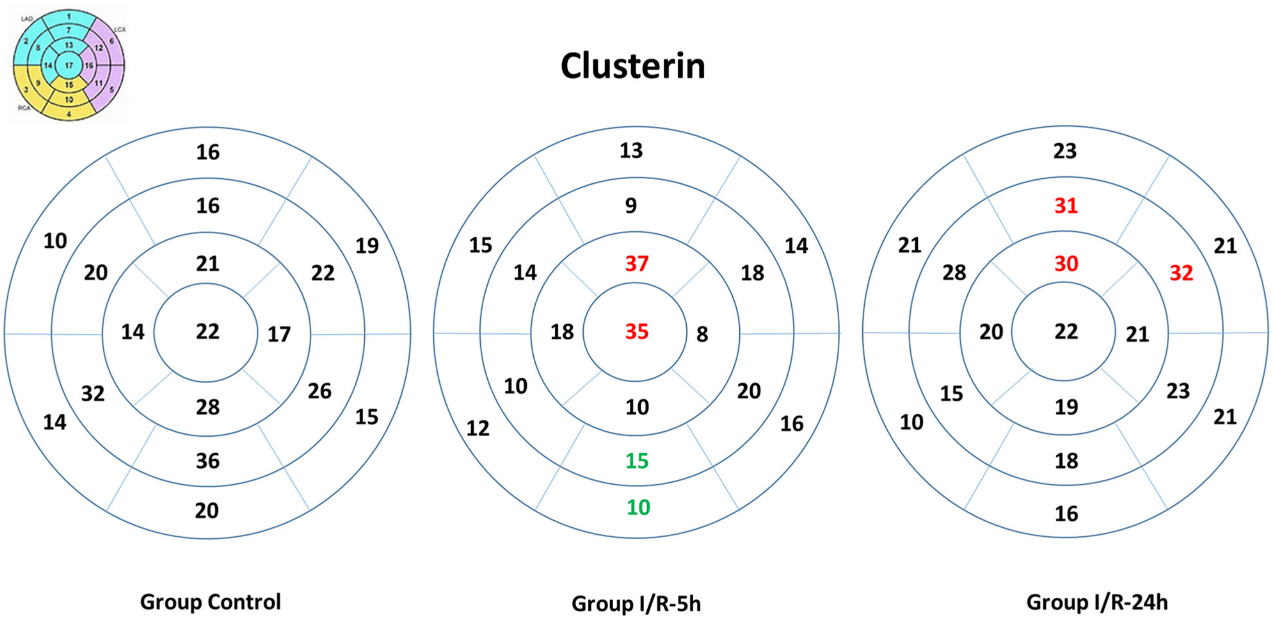

Supplementary Figure 6: Polar map view of relative gene expression values of clusterin. Values with higher expression (differences > 2x s.d.) than the control group in the corresponding segment are red, those with lower expression are green.

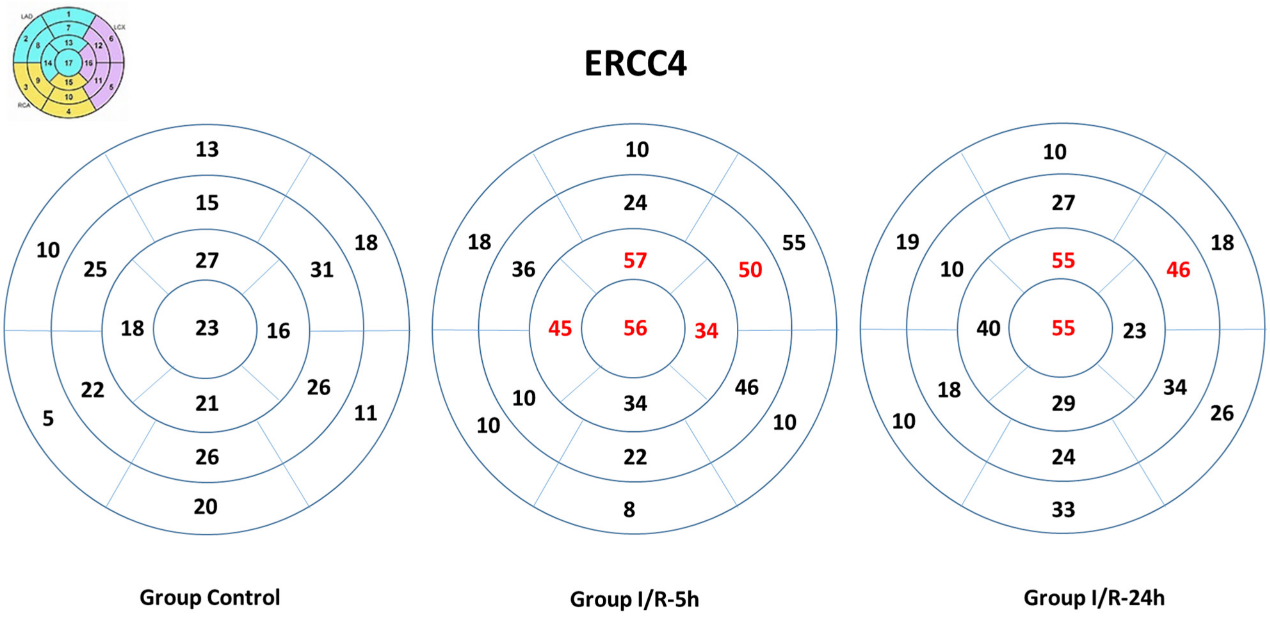

Supplementary Figure 7: Polar map view of relative gene expression values of ERCC4. Values with higher expression (differences > 2x s.d.) than the control group in the corresponding segment are red.

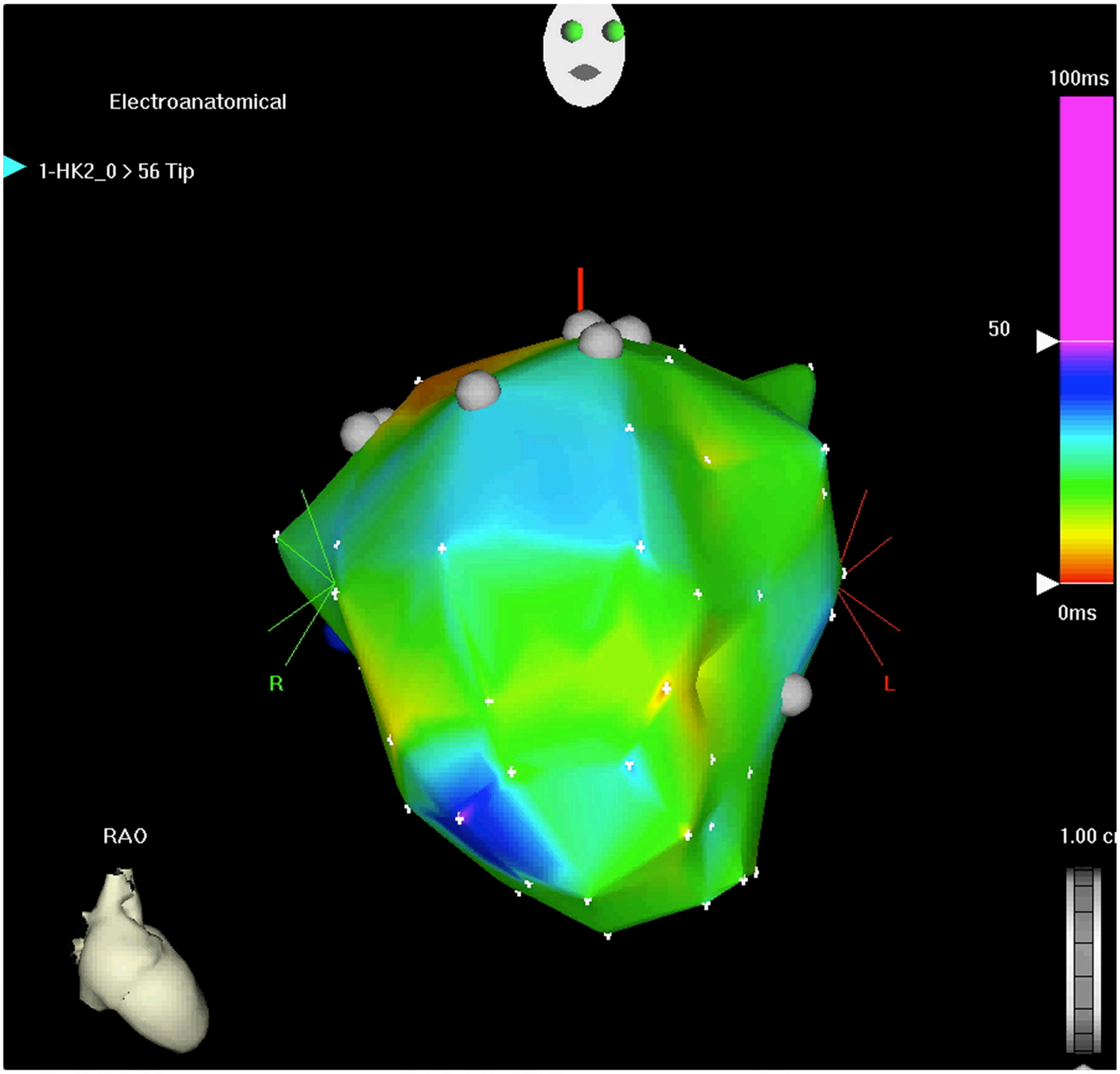

Supplementary Video 1: Online Animations. Timely 3D display of the hexokinase 2 (HK2) gene expression profile of the whole heart - 3D image-omics model. A. Group Control. Timely 3D display of the hexokinase 2 (HK2), which is responsible for coupling extramitochondrial glycolysis to intramitochondrial oxidative phosphorylation and plays a key role in cellular energy metabolism, expression profile, of the whole heart before repetitive ischemia/reperfusion (r-I/R) (Group Control), at 5h (Group r-I/R[5h]) and at 24h follow-up (Group r-I/R[24h]) using the NOGA principles for 3D construction. Three dimensional models were constructed by projecting gene expression fold changes of HK2 on NOGA anatomical mapping coordinates. **(A)** Gene expression pattern of HK2 in Group Control. **(B)** Gene expression pattern of HK2 5h after the r-I/R-stimulus. **(C)** Gene expression pattern of HK2 24h after the r-I/R-stimulus. According to the color coding scheme, pink and blue color represent upregulation of the HK2 gene. Green color represents a baseline value, while yellow and red myocardial areas downregulate the HK2 gene.

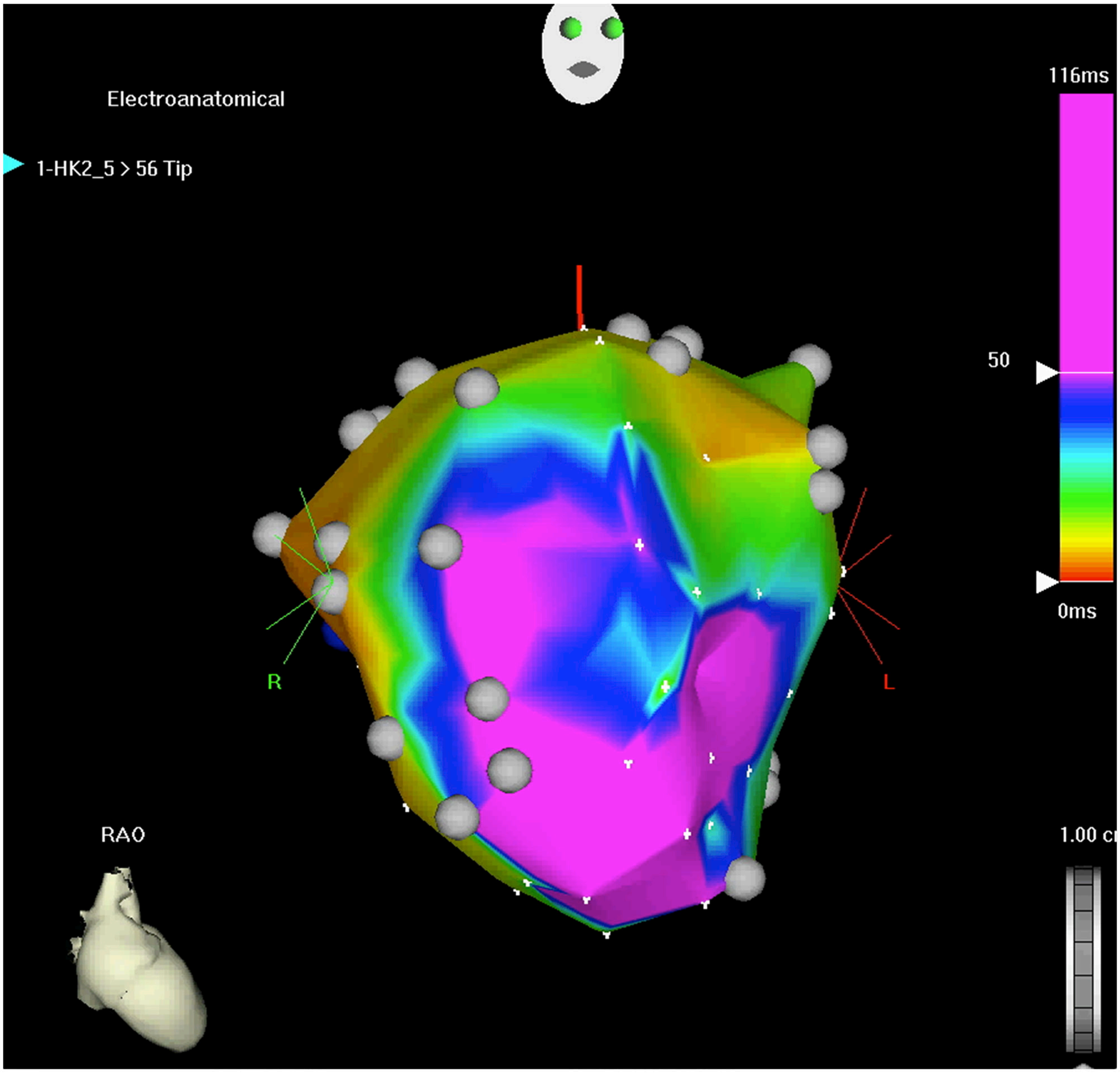

Supplementary Video 2: Online Animations. Timely 3D display of the hexokinase 2 (HK2) gene expression profile of the whole heart - 3D image-omics model. B. Group r-I/R[5h]. Timely 3D display of the hexokinase 2 (HK2), which is responsible for coupling extramitochondrial glycolysis to intramitochondrial oxidative phosphorylation and plays a key role in cellular energy metabolism, expression profile, of the whole heart before repetitive ischemia/reperfusion (r-I/R) (Group Control), at 5h (Group r-I/R[5h]) and at 24h follow-up (Group r-I/R[24h]) using the NOGA principles for 3D construction. Three dimensional models were constructed by projecting gene expression fold changes of HK2 on NOGA anatomical mapping coordinates. **(A)** Gene expression pattern of HK2 in Group Control. **(B)** Gene expression pattern of HK2 5h after the r-I/R-stimulus. **(C)** Gene expression pattern of HK2 24h after the r-I/R-stimulus. According to the color coding scheme, pink and blue color represent upregulation of the HK2 gene. Green color represents a baseline value, while yellow and red myocardial areas downregulate the HK2 gene.

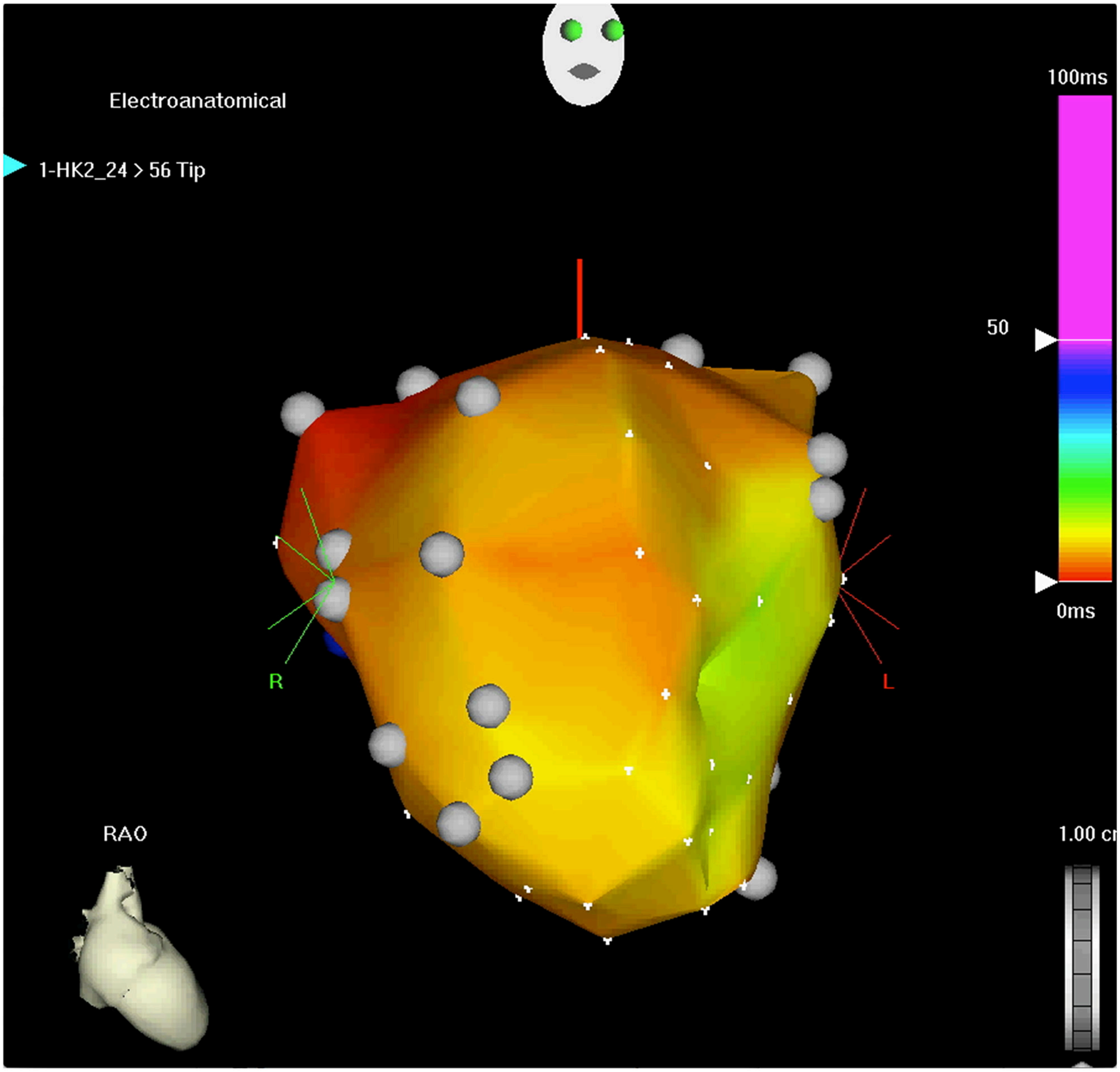

Supplementary Video 3: Online Animations. Timely 3D display of the hexokinase 2 (HK2) gene expression profile of the whole heart - 3D image-omics model. C. Group r-I/R[24h] Timely 3D display of the hexokinase 2 (HK2), which is responsible for coupling extramitochondrial glycolysis to intramitochondrial oxidative phosphorylation and plays a key role in cellular energy metabolism, expression profile, of the whole heart before repetitive ischemia/reperfusion (r-I/R) (Group Control), at 5h (Group r-I/R[5h]) and at 24h follow-up (Group r-I/R[24h]) using the NOGA principles for 3D construction. Three dimensional models were constructed by projecting gene expression fold changes of HK2 on NOGA anatomical mapping coordinates. **(A)** Gene expression pattern of HK2 in Group Control. **(B)** Gene expression pattern of HK2 5h after the r-I/R-stimulus.**(C)** Gene expression pattern of HK2 24h after the r-I/R-stimulus. According to the color coding scheme, pink and blue color represent upregulation of the HK2 gene. Green color represents a baseline value, while yellow and red myocardial areas downregulate the HK2 gene.
